# The “Transparency for Safety” Triangle: Developing a Smart Transparency Framework to Achieve a Safety Learning Community

**DOI:** 10.3390/ijerph191912037

**Published:** 2022-09-23

**Authors:** Paul Lindhout, Genserik Reniers

**Affiliations:** 1 Department of Engineering Management, Faculty of Applied Economic Sciences, University of Antwerp, 2000 Antwerp, Belgium or; 2 Department of Care Ethics, University for Humanistic Studies, Kromme Nieuwegracht 29, 3512 HD Utrecht, The Netherlands; 3 Safety & Security Science Group (S3G), The Department of Values, Technology and Innovation (VTI), Faculty of Technology, Policy and Management (TPM), Delft University of Technology, 2628 BX Delft, The Netherlands; 4 Centre for Economics and Corporate Sustainability (CEDON), KU Leuven, Campus Brussels, 1000 Brussels, Belgium

**Keywords:** health and safety, information sharing, societal merit, TEAM model, true safety

## Abstract

Transparency about health and safety risks is a complex societal, moral, ethical and political concept. Full transparency does not come natural for any of the key stakeholder groups: organizations, authorities and the people. If safety information is not sufficiently shared between them, people and the environment can be harmed. The authors explored the literature on transparency in sharing health and safety information. The findings show that such transparency as a subject is abundant in the literature but the exchange of information is far from complete in practice. Health and safety information is shared both via internal flows within each stakeholder group and via external flows between them. All three main stakeholders in pursuit of true safety for their own reasons, building trust via sharing of health and safety information, require improvement in transparency and a safety information broker between them. This constitutes a smart transparency and information exchange framework. The authors recommend developing a transparency standard, to study cyber-socio-technical systems safety and to include currently underutilized experiential knowledge available from the general public in the societal discourse. The authors propose a societal domain extension to a holistic safety culture model in support of a learning safety community.

## 1. Introduction

Although “shining a light on our errors shows the path to improvement” [1] (p. 1679), acting accordingly is difficult. Transparency about risk and safety is a complex societal, moral, ethical and political concept. At the same time, there are many groups in society with their own take on this subject, e.g., industry, regulators, citizens, employees, activists, experts and media [2]. Since full transparency does not come natural to any of the key stakeholder groups in society, the authors consider transparency about health and safety matters as a fundamental issue preferably to be addressed in company health and safety policies.

Knowledge, power and greed, among other factors, seem at odds with ignorance, truth and morality, among the many factors existing in a democracy [3]. For example, organizations have competitors who might abuse safety information, authorities might withdraw a license to operate or intervene in an organization, hospitals do not like to compromise their reputation e.g., by reporting iatrogenic incidents [4] and people might become aware of thus-far-unknown hazards.

If occupational, exposure or disaster risks are not sufficiently controlled, stakeholders’ roles, economic interests, and vulnerabilities are affected. Organizations’ profitability and continuity, the authority’s environmental protection policies and people’s health can be at stake. Where knowledge and information relevant for prevention of health and safety incidents is not sufficiently shared between these key stakeholder groups, people and environment can be harmed.

There is ample reason to assume this is reality in current health and safety practice. Although the UN endorses a “right-to-know” philosophy in national legislation, not all countries follow this. This means that government regulators and organizations can let their workers and the general public be informed about safety on a “need-to-know basis” in, e.g., chemical, nuclear and building industries [5,6,7]. This illustrates that there is no fully open information exchange within an organization about safety matters for a variety of reasons, e.g., that a piece of information is considered as not relevant to workers, regulators or the general public, or not is not being shared for commercial reasons.

An indicative example of this is that some 21 reasons were found as to why information about language-related accidents is difficult to find and use for improvement in prevention activities [8]. This indicates that organizations, governmental institutions and the people—workers and general public—each have their own reasons for not fully sharing accident-related information. Even at the national level, credibility, truth and openness are the subjects of polarized debates and crucial for trust by the people in democratic countries [9].

This is because organizations do not all have sufficient risk appetite, are fearful that competitors will discover their trade secrets, or are hesitant to alert the authorities or the general public. All of this leads to incomplete and partially undisclosed risk inventories [10]. In such cases, moral transgression can also adversely affect the safety of workers, the general public and the environment [11].

Nonetheless, both organizations and the people need to exchange information about health and safety matters, although this is for different reasons. The people need work and confidence in continuity, health and safety. Organizations need acceptance in society by government and the people. Organizations that are open and transparent to inspection from authorities and the people can be prevented from moral transgression in health care and in industry [12]. Furthermore, disclosure of health and safety information can have a positive effect on company profitability [13]. Exchange of important information and application of safety-related knowledge are key to safeguarding expertise and to organizational learning. Although a large amount of such experiential knowledge exists, there are also challenges to learning aimed at applying critical epistemic or technical knowledge and the right skills in the face of a safety threat requiring immediate response. Rare, unstructured problems, uncertainty, information overload and time pressure all require such knowledge to be readily available [14].

Providing an overview of where information channels are located, the stakeholders in society these channels connect, and the completeness of the health and safety information that is actually exchanged can be a daunting task. Therefore, a means to explore the exchange of health and safety information on a general level is needed. The purpose of such an overview is to gain insight and enable or enhance prediction of the system or the phenomenon being studied [15]. Using “transparency” as an indicator for completeness of the information exchange, the authors set out to construct a health and safety information flow framework, and to add a societal domain extension to the holistic TEAM safety culture model, which connects organizations’ internal aspects of safety and their mutual relations [16,17]. These proposals are in support of the search for “true safety” (defined here as safety as audited, as perceived within the organization and as felt by individual workers) and of “transparency improvement” in an organizational—and even societal—health and safety area. This study centered on the following main research question:

How can organizational health and safety information be shared in a more transparent way in society?

In search of an answer, the authors investigate several sub-questions:

- How can health and safety information exchange between stakeholders be mapped on a general level? (Location of risk, health and safety information flows, stakeholders, internal/external, flow mapping, see Section 3.1).
- Which aspects can play a role in this exchange of information in practice? (Flaws, transparency definition, see Section 3.2; organizations and societal merit, see Section 3.3; stakeholder perspectives, true safety, benefits, see Section 3.4).
- Which best practices currently exist? (See Section 3.5).
- Are all types of safety being considered? (e.g., real, audited, perceived, true, and regulated safety, see Section 3.1, Section 3.4 and Section 3.7).
- How can such sharing be embedded in safety culture? (Emerging challenges, TEAM model extension, see Section 3.6 and Section 3.7).
- What can be said about moving towards a learning health and safety community of practice? (See Section 3.8).
- Which standards are considered relevant to the cause of transparency in safety information? (See Section 3.9).

## 2. Materials and Methods

Since the aim of this study was to explore the subject matter, rather than to perform a systematic review, the authors chose the scoping review method for searching and selecting sources [18,19]. Literature about “transparency in health and safety information” can currently not be considered as a structured scientific field and does not constitute a body of knowledge [20]. No specific databases are available on this particular field. The scoping review method is flexible and allows gradual development of the set of search terms as the search in literature develops.

Initial searches were used to compose a set of search terms. Then, a series of searches with different search terms combinations was conducted. Finally, auxiliary searches were added to address specific subjects encountered. First, a stakeholders and health and safety information flow map was constructed on the basis of the first sub-question findings. Each admitted source was then screened on themes related to the main research question and sub-questions. The sub-questions and theme findings were used to progressively structure the results section into sub-sections. Then, in a qualitative meta-synthesis process, the theme descriptions were successively refined and detailed as more sources were included [21,22].

Since exploring a wide range of societal and scientific fields was required here, Google Scholar, ResearchGate, Academia and their associated databases were used.

Relevant scientific sources, available in English, originating from industrialized societies and published from the year 2000 onwards, were admitted. Several sources published earlier were admitted because of their bearing on the subject at hand. Moreover, several non-scientific sources were admitted because of their particular relevance for this study [23,24,25].

Two preliminary searches were conducted. The first, on 9 December 2021, searching Google on “Understanding transparency for Safety”, resulted in 424 million returns. Focusing on scientific sources only, a second search on “Transparency and safety information” in Google Scholar on 12 January 2022, resulted in 1.11 million returns. As a subject, the transparency of safety information appears to be abundant in the literature. A large proportion of the sources returned is about transparency in food and drugs safety for consumers [26,27]. A smaller part of the sources returned is about product safety for consumers and deals with liability issues. Sources in these categories were further excluded in this study as they are not about transparency itself.

To narrow down the search yield even further, a set of search terms was extracted from the sources found in the initial searches. These were: “communication, community, credibility, culture, health, industry, information, learning, process, risk, safety, transparency, truth, understanding, organizational learning, true safety, best practice, knowledge exchange”. These terms were then used in several combination searches.

**Figure 1:**
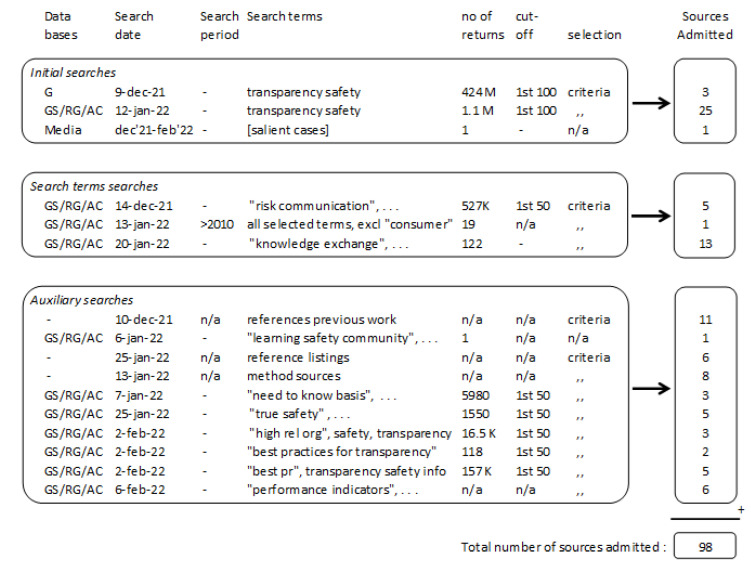
Search process and results. (G = Google; GS = Google Scholar; RG = ResearchGate; AC = Academia).

Finally, several auxiliary searches were conducted to explore specific subjects encountered when the search process results unfolded. This search process [19] and the resulting admission of a total of 98 relevant sources are depicted in Figure 1.

## 3. Results

The literature sources were screened on several subjects: where safety information is exchanged, what it is about, which stakeholders participate, whether the information flow is internal or external, which flaws are observed, how transparency is defined, which drivers support transparency and which best practices are being reported.

### 3.1. Location of Information Flows

#### 3.1.1. Types of Risks

In safety management practice, risks are usually divided in different types: occupational risks, exposure risks and disaster risks. Occupational risks are well understood, frequently occurring and easily observed; and can happen anywhere at work, have acute consequences for workers and affect groups of people in society as a whole. Exposure risks are continuously present in the daily working and living environment; have unknown, insidious chronic effects on individuals, groups of people, the environment and society; and are difficult to observe. Disaster risks however, are not always well understood, are rare, can happen in specific situations in industry, and can have a huge and acute impact on individuals, groups of people, the environment and society.

#### 3.1.2. Stakeholders

In many societies, risks are controlled by organizations and regulated by government authorities. Since risks originate from activities in an organization, the risk inventory and control, risk communication and safety management activities reside there. The authorities provide a license to operate, act as independent regulator and mitigate when things go wrong, e.g., in case of a disaster. Although some individual persons might be employed by an organization or a government institution, people are regarded here as a separate collective. People are the vulnerable stakeholder since any negative effects coming forth from the risks will primarily affect them.

Apart from these three main stakeholders, there are several others in society related to safety and risks. These are the media, taking part via, e.g., investigative journalism, exposure and publicity about abuse and misconduct; the scientific institutions, taking part via, e.g., safety research, risk assessment, method development and analyses; and the engineering companies, e.g., via providing designs for processes, installations and control software. Since these other stakeholders are not directly being held responsible for risk control, we explored transparency on the following basis: all communication about risk and safety takes place within the triangle of main stakeholder groups: organizations, authorities and the people. We distinguish two types of information flows: internal, i.e., within a stakeholder group, and external, i.e., between stakeholder groups. We assume each of these flows is bi-directional.

#### 3.1.3. Internal Safety Information Flows

Organizations

Within a single organization, three components, usually jointly named safety culture, determine the safety-related information exchange. These are: (1) personal psychological factors, such as risk attitudes and skills; (2) risk perceptual factors, referred to as safety climate, such as management commitment, leadership and trust; and (3) observable factors related to people, procedures and technology [28].

In the context of The Egg Aggregated Model (TEAM), a safety culture model, these factors are assigned to three safety aspects, distinguishing audited, perceived, and real safety [16,29]. In this model, audited safety is about observable factors in an organization in relation to safety. Perceived safety is about shared perceptions within the group of people involved, referred to as the safety climate. Real safety is about the minds of the individuals and their intention to behave with respect to safety [16,29,30].

Safety information is being exchanged in many ways within a single organization, e.g., between the safety culture domains, between departments, between production sites, with suppliers, and between management and employees. Such information is also shared between organizations, on, e.g., bilateral, cluster, branch, sector, regional or international geographic levels. Doing so, organizations exchange safety-related knowledge, e.g., near misses, lessons learned, risk assessment knowledge and know-how, and codes of good practice. This results in various internal organizations stakeholder group safety-related information flows.

Authorities

Inside the authorities’ stakeholder group there is an exchange of risk and safety-related information with international organizations, e.g., the European Council (EC), the United Nations International Labor Organization (UN-ILO) and World Health Organization (WHO), which can lead to newly identified hazards, new standards, better practices and new legislation. On a national level, the risk and safety domain touches upon the responsibilities of several ministries and a range of public institutions which are connected via a suite of consultative bodies, and are under political control. In this structure, the health, safety and environment aspects of risk are subjected to governance and require often complex, case by case ethical trade-offs and decision making [31]. Organizations face two separate sides of the authorities: the negotiation, coordination and permits side, and the regulator and law enforcement side.

The people

The people stakeholder group is split over many groups, e.g., citizens, employees, foreign workers, general public, patients in care institutions and several other groups, each with their own societal position and interests. Between these groups there is limited information exchange relating to risk and safety. This exchange takes place, e.g., via the public media, unions, patient associations and professional associations.

#### 3.1.4. External Safety Information Flows

Information about risks and about safety is not only exchanged within, but also between, the three stakeholder groups.

Organizations

Organizations can share risk information in a proactive manner on a general level (e.g., information to the general public), on a specific level (e.g., emergency drills, giving information about a workplace, or a safety instruction for a specific hazardous activity), in a proactive manner (e.g., informing employees about risk assessment), or in a reactive manner (e.g., crisis communication to the general public, or giving hazardous chemical information to a fire brigade squad team during disaster mitigation).

Organizations provide information about, e.g., profit, shares, products and services, and production processes to the authorities [32]. Organizations also disclose information about their supply chains for several reasons. Authorities may impose trade restrictions and taxes, and the general public and business-to-business customers may want to choose on the basis of preference, e.g., for a country of origin of a product, compliance to testing requirements and product performance-oriented standards, e.g., for autonomous robotic systems [33]. These can be related to, e.g., product quality, the absence of child labor, safe work in remote production plants, and sustainability or environmental impact [34]. Safety information about products and services is supplied to customers. Organizations provide information about risks and about health and safety, both to the authorities and to the people; in the latter case, this is provided to employees and foreign workers on an individual level and citizens on a collective level. Health and safety information provided to government regulators, e.g., during inspections and audits, consists, in part, of performance indicators. Such indicators reflect, e.g., the general state of affairs on health and safety in an organization via measuring regulatory compliance, emergency response, ethical performance, waste reduction, worker training activities and community involvement [35]; the safety of processes via measuring process conditions, equipment status, activity monitoring and effect detection [36]; and safety management system performance via measuring personnel training and behavior, risk assessment, adequacy of safety provisions, safe work practice, change control, emergency preparedness, monitoring and analyzing unwanted events, and management review activities [16,37]. However, indicators for transparency and disclosure are rare.

Organizations receive regulatory information from the authorities, e.g., legislation, license to operate, inspection reports and enforcement activities.

Health care organizations, e.g., hospitals, inform patients about risks and can disclose information about medical errors [1], and caregivers can both exchange information and be held accountable in a supportive culture [38]. This is better than to engage in a legal confrontation with their patients [4]. Furthermore, disclosure shows whether there is respect for ethical principles and the knowledge obtained enables organizational learning from accidents [39].

Occupational Safety and Health (OSH) information, often referred to as OSH reporting, is provided by companies for three reasons other than legal obligations: it supports their business (e.g., by showing a good track record), it is a way to be accountable (e.g., by reporting incidents) and it helps to create an image in society (e.g., by offering a safe work environment) [40]. The exchange of OSH data on a global scale can reduce the harm to people [41,42]. Many well-proven ways to share health and safety knowledge exist [43,44]. Such knowledge empowers citizens and workers, allows more choice by the people and adds a democratic political dimension to transparency [45].

Authorities

The authorities both provide information to and receive information from organizations. Depending on the industrial sector and the magnitude of the risks involved, an organization must comply with specific legislation, properly inform the authorities, meet regulatory risk criteria and ensure that residual risks are accepted in society.

Ideally, the authorities also provide safety-related information to the people. Some of this is made accessible for the general public, e.g., on public health via inspection reports [46]. The general public receives safety-related information from the authorities, e.g., via occupational accident statistics, via public reports generated by non-governmental organizations (NGOs) and government institutions, and via crisis communication [47]. Governments also act on the international level, e.g., in a bilateral context, act within economic communities, and act outside national jurisdiction, e.g., in off-shore and sea-bed mining [48]. Governments internationally engage, e.g., in crime fighting, in harmonizing legislation and in treaties about safety and standards. Implementing adequate transparency in all these areas is a major challenge and subject of debate [45,49]. In the past decades, e.g., risk maps were placed online for the general public in The Netherlands, but whether this leads to better informed, empowered and more involved citizens is not clear [50].

Safety-related best practices are converted to standards which are negotiated and agreed with the industry and health care sectors. Government legislation and court rulings are made available to organizations and the general public. Ideally the authorities are receptive and knowledgeable, and maintain a level playing field for risk and safety matters. In practice, governments formulate priority policies to match actual effort for regulatory and law enforcement tasks with, e.g., current political priorities and available capacity in authorities’ institutions. Safety as intended from a government point of view is therefore to be considered separately from regulated safety.

All stakeholders together—organizations, authorities and the people—could strive to achieve the highest feasible safety level: true safety. Worldwide, the authorities have limited means to ascertain true safety and rise above mere audited safety. In some cases and countries, an increasing emphasis on safety culture during inspections demonstrates the ambition to go beyond the observable part of safety culture [51,52].

The people

This stakeholder consists of people with different roles, e.g., citizen, general public, employee, worker, foreign worker, consumer, patient, care provider, representative or politician. The general public hardly interacts directly and structurally with authorities and organizations when providing and receiving safety-related information. Noted exceptions are the workers having a collective say via unions, works councils or workforce participation [53]. Also noteworthy is the increasing omnipresence of cameras among the general public, which is now being matched with police officers wearing body cams. Here the people want protection against excessive police violence and police want to prevent perpetrators hiding in a crowd [54]. In democratic countries, citizens can express their general preferences via elections and collective political action channels. However, in practice, industrial and institutional safety has little or no presence in the societal and political debate [55]. For specific situations on the scale of a village, town or city, people can have a say in the local political arena, e.g., when their environment is affected by planned new industrial activity. This does not always explicitly address health and safety issues for the general public and the workers, however.

Specific employees in an organization, e.g., the safety engineers and safety managers, are able to contribute to external information exchange about safety. They can do so, e.g., in professional associations, in corporate safety seminars and in routine safety monitoring data exchange.

Generating knowledge and providing safety-related information by the people currently happens in an indirect and haphazard manner. Examples of this are court cases, investigative journalism, media exposure, scientific reports, complaints, demonstrations, videos of police officers exerting violence and citizens’ political action. Hence, the people, possessing a much-underutilized experiential knowledge about health, safety and wellbeing, are informing both authorities and organizations in a variety of unsolicited and unstructured ways.

**Figure 2:**
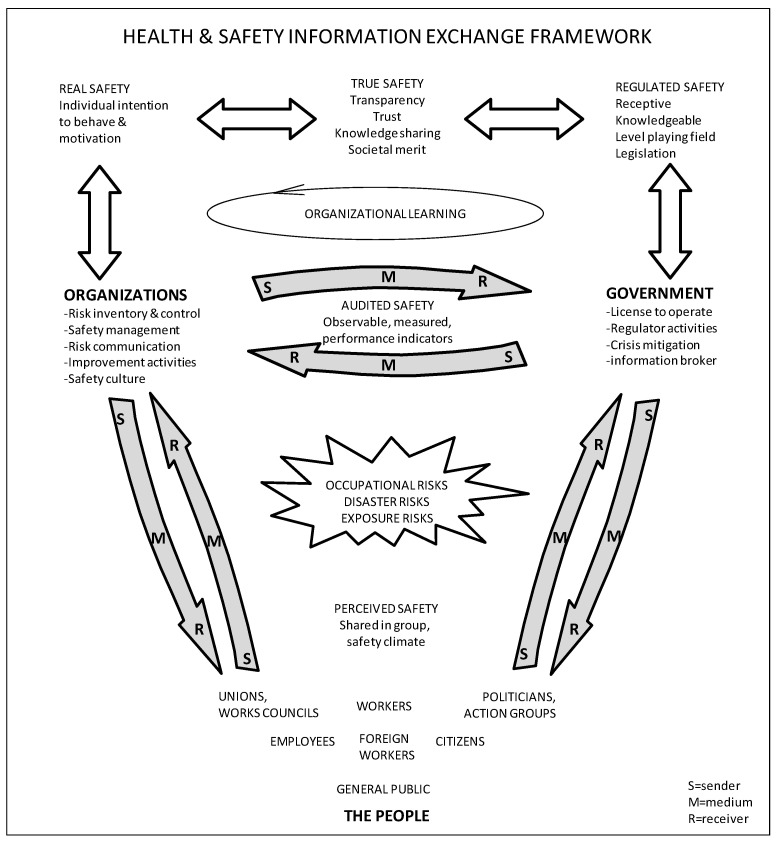
The “transparency for safety” triangle, a health and safety information exchange framework, showing information flows between stakeholder groups.

Exploring health and safety transparency requires a framework that includes both the internal information flows within each stakeholder group and the external information flows between the three stakeholders. A triangular-shaped health and safety information exchange framework emerged; see Figure 2.

The authors constructed this framework which maps the health and safety information flows; supports their transparency to be located, assessed and improved; shows the context in which knowledge sharing, organizational learning processes and societal merit can be evaluated; and which enables health and safety improvement proposals to be appraised and executed. This framework includes the continuous pursuit of true safety by all three main stakeholders in their joint endeavor to create a learning safety community [56].

### 3.2. Information Exchange in Practice

#### 3.2.1. Flaws in Information Flow

To be able to investigate transparency, it is not only important to know where the information flows are, but also in what ways each of the flows can be affected. There are limitations, shortcomings and even deliberate mishaps affecting the quality of shared risk information between stakeholders in society [43,44]. Examples of this are found in a range of areas in society, e.g., the safety of patients being treated or transported outside hospitals appears to be significantly underreported [57,58], language problem-related accidents are underreported in industry [8] and the knowledge about mining waste management has “… become deeply corrupted by economic interests and made inaccessible due to political, organizational and disciplinary silos and schisms.” [59] (p. 123).

A common example is that employees are informed about health and safety matters on a “need-to-know basis” [7] (p. 23), suggesting that, e.g., perhaps even for their own safety, they might need to know more than is actually shared by an organization.

Other important flaws can be hidden in the method of knowledge transfer to people. The extent to which such knowledge reaches the targeted group of people can be affected by, e.g., illiteracy, foreign language, superficiality, overrated information absorption capability [60], end users not being involved, and other quality of information and communication issues [44].

Authorities can be misinformed about risks or about their potential effects, for example, by omissions or incorrectness in the inventory of risks provided by organizations [10]. Organizations hardly exchange proactive or strategic safety information in support of the development of a learning safety community.

Changing such situations can require considerable effort, e.g., the implementation of the International Civil Aviation Organization (ICAO) Global Aviation Safety Roadmap to further reduce the fatal accident rate per million flight hours [61].

Several free information flow-constraining legal issues are reported in the literature. These are secrecy, privacy and discrimination [62]. Methodical issues exist relating to performance measure definition, data composition method, reliability and validity [63]. There are also issues such as terrorism vulnerability [50], court systems prohibiting disclosure of case information and cost impact to consider. An example of the latter is the administrative burden associated with information sharing which affects hospital efficiency of core medical activities, e.g., by external supervisors not operating in a coordinated way [64].

The general public may be unaware of their being subjected to emission of hazardous chemicals and of their living environment being polluted, e.g., by preventing signals reaching the public media. The media can downplay or exaggerate information and distort the view of the general public [65].

The Internet is playing an increasingly important role relating to dissemination of health and safety information [43,44,60]. The reliability of such information is not guaranteed, however.

#### 3.2.2. Transparency

Such flaws in safety communication comprise more than simply a reduced information transfer.

The consequences of such flaws can cause trustworthiness issues and credibility problems, and can affect the corporate image in a negative way. Moreover, the efforts to establish a safety learning community are jeopardized.

People can manipulate information in many ways. Information can be, e.g., not gathered, withdrawn, incomplete, biased, distorted, delayed, faked, leaked, inaccessible, deemed confidential, spread selectively or lied about, just to mention a few of the possible ways. The authors contend the term transparency is more appropriate here. Several definitions of transparency exist.

Definitions of transparency are first of all about information exchange and visibility of the inside of something from an outsiders’ point of view [54]. Less often a second aspect is also implicated: people’s “right to know”. The definition is not about how transparency is provided, obtained or enforced. From a health care point of view, transparency can be defined as “the free, uninhibited flow of information that is open to the scrutiny of others” [66] (p. vii). From a phenomenological point of view, the information flow between a subject individual to a researcher can be incomplete, distorted or incorrect [67]. From an information transfer point of view, the ethics of information transparency are important [68]. From a knowledge transfer process point of view, there are many factors to consider, such as first describing the problem and context, which are then leading to specific knowledge, which in turn triggers intervention and usage [69]. In such processes, knowledge is modeled as being channeled from a source via a knowledge broker to a user [60,70]. From a communication point of view, information can be transmitted through an imperfect communication channel from a sender, via a medium, towards a receiver in one direction [71]. From a physics point of view, reduced optical transmission, translucence and see-through qualifications as “opaque” or “transparent” are considered as properties of an object or a medium.

In social and economic contexts, the terms insight, openness, accountability, disclosure, transpicuousness and clarity all represent constituent parts of the meaning of the term transparency, thereby assigning properties to both sender and medium.

In this study “transparency” is defined as “the degree of health and safety-related information transmission within and between stakeholder groups”. This assumes a flow in both directions and includes the communication theory (S)ender, (M)edium and (R)eceiver aspects (see Figure 2).

### 3.3. Moving Up the Societal Merit Ladder

Thinking about why organizations would need transparency in the first place, the authors considered their role in society in relation to transparency about what they do.

A lack of transparency disturbs the trust within and between the stakeholders [6]. The main cause is insufficient information exchange, casting doubt about, e.g., intentions, quality of received information, completeness of accident statistics, and safety itself, such as the proper control of ultrafine dust exposure risk. Such trust is built on national cooperation between governmental institutions for regulatory tasks and law enforcement, and by adequate and sufficiently visible safety measures in organizations, supported by adequate information exchange [6]. Lack of trust can be a threat to the continuity of an organization [7]. Organizations need to respect the right-to know of citizens and workers [45]. Otherwise, organizations can, e.g., become the subject of societal controversy and government intervention.

Although the original corporate social responsibility (CSR) thinking was depicted as a pyramid, with at its top “being a good corporate citizen” [72] (p. 42), and thus lining up with Maslow’s hierarchy of needs pyramid [73], the authors contend that organizations, in addition to being “good” at achieving their goal, at the same time also aspire to a participative, appreciated and valuable role in society. Organizations take a series of steps to get there. Organizations can simply exist, be compliant to legislation and pay taxes. Organizations can present themselves in various ways to impress customers and share information in support of the corporate image in the marketplace. Organizations can also engage in societal partnerships to develop, e.g., a kindergarten, a local fire brigade, or an industrial area, support refurbishment of living quarters in a local community, or even take the lead in a project. Therefore, the analogy with the citizens’ participation ladder [74] is also relevant here. Organizations engage in roles with societal merit [75].

For organizations there appears to be a societal merit ladder, originating from a good product or service, accompanied by good safety management, transparency and credibility, and underlined by a license to operate. In turn, this enables an organization to undertake business transactions and make a profit, provide people with jobs, and then build up trust, proceed to an acknowledged and well-supported safety culture, and open the gateway towards sustainable business and a good corporate citizenship-inspired, responsible, respected and influential role in society [11,72,76].

Finally, an organization has the possibility to become part of an “organizational learning of safety community of practice” [56] (p. 7). Although a start-up organization may want to attempt leap-frogging this path, securing every next step appears to be necessary [77]. The relation between any step on this ladder and the next one is complex. Although each preceding step on the path up this ladder is regarded as a necessary condition for reaching the next step, underway there may also be other rational, emotional and crucial factors to consider. Sufficient transparency about safety matters is a means of communication supporting trust and credibility.

Organizations aspiring to a higher level on the societal merit ladder need to acknowledge the right-to know of citizens and workers, to improve knowledge sharing, increase transparency, embrace moral and ethical principles, follow CSR guidance and become a member of a learning community. Organizations need to move upwards on the societal merit ladder in a sustainable and effective way [78,79]. The areas of ethical conduct and corporate social responsibility are interconnected and require dedicated management attention [76].

Organizations therefore also need to minimize the differences between the real, audited, regulated and perceived versions of safety in the TEAM model and seek true safety. Hence, any attempt to improve transparency in safety communication would necessitate consideration of all information flows and allow all upwards steps in the societal merit ladder to be taken.

### 3.4. Different Stakeholder Perspectives

Transparency is being looked at in different ways. The people experience a lack of information and are not directly involved in a societal dialogue about health and safety matters. The authorities work with a balance since a government can provide both too much and too little information [32,54]. Organizations have traditionally provided little information but can gain trust by sharing more and by increasing their societal role. All stakeholders are in pursuit of true safety, each for their own reasons. Best practices are relevant to all those who work in support of this endeavor.

#### 3.4.1. People’s Transparency Need

The people, both the workers and the general public, need confidence in what organizations do, in continuity of their personal dealings and in protection of their personal health and safety. People need disclosure to be dependable in the future. People also need choice, e.g., disclosure of information about workplaces to enable employees to choose safer and more socially acceptable organizations, although this is not a common regulatory requirement [32].

Health and safety information reaches the people, at home and at their workplaces, from two directions: from the authorities and from organizations. Health and safety knowledge transfer between the sources and the users of such knowledge is the complicated key issue here. Due to, e.g., unidirectionality, mismatches between speaker and audience, and ineffectiveness of the knowledge broker, learning is impeded, both in industry and in health care [42,70,80,81,82,83]. Apparently, a government needs to ensure that safety knowledge or, more generally, safety information is exchanged via a safety information broker [70].

The public and professionals may differ significantly in their view on health and safety risks, which is a further reason to engage in dialogue [55,84]. For instance, in health care, patients need information based on suitable performance measures, backed-up by standards and safeguarded by an institution. Transparency improves health providers’ performance and allows care consumers to have a choice between providers based on quality and cost [63].

#### 3.4.2. The Governmental Transparency Balance

Can the authorities be fully open? This question has led to much debate since there are both reasons to respond to the individuals’ desire for transparency and reasons for secrecy, discretion or confidentiality in several areas. Secrecy is associated, e.g., with vulnerability for terrorism, national security, law enforcement, proprietary information and personal privacy. Moreover, high costs for sharing of all governmental information with the general public are a limiting factor. Openness as a principle is associated with, e.g., democratic accountability, whistle blowers’ protection, government officials fiscal and budget information, people having the right-to-know, and safety.

One can debate secrecy and when it is justified [32]. Most of the objections against disclosure originate from companies, e.g., concerning salary levels, work conditions, proprietary information, trade secrets, commercial information, key personnel, workplace safety, accidents at work and labor contract conditions. Not all these objections are legitimate, however, and some of them may go against the interests of the general public, the government and individual employees, e.g., hygiene ratings in restaurants, exposure monitoring data at workplaces or regulatory safety inspection results. There may be too much workplace information to share and it may be too technical to be understood, so “targeted transparency” [32] (p. 377) may be necessary.

A recent survey among food safety regulator inspectors in The Netherlands shows that they perceive more government transparency as better regulatory performance [54,85].

All this leads to a governmental balance between confidentiality and openness [86,87]. The governmental balance between opaque or closed, and transparent or open, must be found in a dynamic environment [54]. This implies that a “right” level and focus of transparency must be chosen, and that not all of the stakeholders may agree to this choice in all cases.

#### 3.4.3. Organizations

The core problem of transparency can be clarified with: “Transparency lies at the intersection between the public’s right to know and corporation’s right to privacy” [88] (p. 77). Not only are reactive and obligatory transparency and disclosure essential for corporate governance in order to allow informed decision making by the financial partners involved [88]. A corporation should provide the truth, do this in a proactive manner, and address all stakeholders including the general public. This ensures accountability and opens up possibilities for control by the board, shareholders, external stakeholders and the government. Several elements to build an organizational culture of transparency are proposed, focusing on truth, letting people speak, stimulating debate, enabling multiple information sources and admitting mistakes. In this way, a learning community is formed and fostered. Information quality must be safeguarded in terms of accuracy, completeness, relevance, timeliness and accessibility.

#### 3.4.4. In Pursuit of True Safety

Organizations, governments and people share a common objective: the pursuit of true safety. This concept, also referred to as a true safety culture, is about reaching the best attainable safety level. The path follows a series of steps, first described in the 1990s [89]. These steps are Pathological–Bureaucratic–Generative, and relate to how organizations deal with safety-related information. Later, two additional levels were proposed [90], leading to the safety culture steps being used today: Pathological–Reactive–Calculative–Proactive–Generative [91]. The evolution of safety culture coincides with information sharing and trust [51,52]. Both of these increase when climbing the steps towards a true generative safety culture, the end level to be reached after a long history of safety culture development. The associated safe work values—perfection, ultimate and ideal—are fully internalized and true information is exchanged between management and workers.

An organization needs to engage with true safety since workers, general public and environment not only need the bare minimum protection as required by law. An organization also needs a good track record and acceptance in society in order to be able to continue doing business.

The government needs to engage with true safety when appraising risk assessments [10] and when deciding on a permit or a license to operate. Regulatory activities need to be geared towards stimulation of organizations to build generative safety systems [52].

Empirical evidence shows, however, that poor measuring tools for safety culture and for monitoring of adverse events cannot be used to predict the true safety of patients in hospital organizations [92]. An earlier study in multiple hospitals indicated a correlation between a more positive patient safety culture and fewer adverse incidents, however [93].

True safety is referred to as a goal in the TEAM safety culture model [16,29]. This model distinguishes three types of safety: real safety as intended in the heads of employees, audited safety in the inspection reports of the authorities, and perceived safety as it is shared and felt in the safety climate of an organization.

#### 3.4.5. Benefits from Transparency

If all three main stakeholders pursue true safety for their own different reasons, improved exchange of health and safety information via the channels between them would clearly also require more transparency between them. So how could each of the stakeholder groups benefit from improved transparency?

Organizations benefit from opportunities to learn about risks, health and safety matters from each other, build credibility and trust, proceed along the societal merit ladder and move towards a safety learning community. Inspired by CSR, organizations need to improve and appraise their own transparency, assess their own position on the societal merit ladder and use newly designed performance indicators.

The people will benefit through improved transparency from organizations and government and will be enabled to make better choices in support of their health, safety and wellbeing. People also need confidence in continuity, and need to be heard in society when things go wrong or in cases where they are at a disadvantage, e.g., due to unethical practices. People need to be accurately informed, need to have a say regarding risk, safety and health, and must be allowed to bring their experiential knowledge to the table in an acceptable way.

The government can benefit from transparency in their regulatory role, their choice of an appropriate governance focus, and their decision making on law enforcement methods and priorities. The development of a safety and transparency standard can facilitate the set-up of a safety information broker which is acceptable for all stakeholder groups.

### 3.5. Best Practices

Several best practices were found in the literature. Not all of them are readily available to all stakeholders, however. Moreover, not all of the aspects of transparency are covered. We have grouped them on basis of similarity:

- Intermediate organization

Corporate CSR, transparency, health and safety information exchange, and organizational learning would best be supported by an intermediate organization [56,70]. In this way, knowledge can be exchanged and stored centrally, ensuring equal information for all stakeholders.

- Indicators

Various indicators could be used to monitor the transparency performance of a government system: are practice and policy in line, are stakeholders engaged, are they actively implementing, are support and evaluation activities being undertaken, and is there transparency throughout the process [94], willingness to accept public scrutiny and dialogue, and acceptance of a quality of evidence standard [95]. However, these indicators are not easy to measure.

- Safety culture

Transparency is taken up in industrial safety culture as an aspect of internal safety management system communication, situated among the perceptual factors of the safety climate. This is important for, e.g., risk identification, risk awareness, risk perception, safe work procedures and crisis communication [16,29]. Regarding patient safety, an internal culture, supporting and prioritizing transparency and safety, is better for patient safety, and a link to human resources management and employee performance is recommended [38]. Regarding patient safety in the UK, the focus is on four aspects of transparency: clinicians–patients (about errors), between clinicians (peer review, info sharing), between health care organizations (collaboration), and clinicians and organizations–public (quality and safety data reporting) [66]. The three most common practices to achieve patient safety culture improvement were identified as goal/planning/leadership support strength, use of well-known prior safety initiatives, and frequent measurement of culture and wide dissemination of the findings [96].

- Sharing information

Key areas for public trust, e.g., in food research, are openness about all phases of research, the process, stakeholders involved, funders, beneficiaries, strength of evidence, efficacy, opponents, conflicting interests, and biases regarding, e.g., safety. Sources of mistrust are inaccurate, false or weakly supported information, unachievable expectations and unproven product claims [95]. Governments and non-profit organizations are generally willing to share their financial data. Although open sharing with the general public of financial information, commercial data and evidence may be difficult for organizations in a competitive environment [3,97], the disclosure of information about products, services, production processes and governance may help to “press firms to reach beyond compliance” [32] (p. 351) regarding labor legislation.

- Cooperation

Cooperation and transparency among chemical plants sharing space in a chemical industrial park is recommended for hazard management [98].

- Communication

Improvements in construction process transparency, and the ability to communicate with the people involved in it, ensure that safety problems, deviations and unnecessary waste can be prepared for and are corrected [99].

- External verification

Information systems should be designed and built to be able to provide transparency about how information is retrieved, where the data originate from, and which stakeholders and which users are involved [100]. In addition, external parties should be able to verify the systems’ correct functioning. In practice, there are several major obstacles, e.g., privacy of users, the right to be forgotten, systems intricacies and business confidentiality. People have the right to know about automated decision making that affects their daily life. Ethical aspects are also relevant, e.g., whether consequences of such decisions are fair and who can be held accountable.

- Learning for safety

Transparency was at the basis of a safety improvement project in international civil aviation. The exchange of audit results with the general public led to the Global Aviation Safety Roadmap and improved cooperation between stakeholders [61].

### 3.6. Emerging Challenges in Safety Culture

Beyond the “classic” audited safety and workplace safety improvement objectives, as addressed in the holistic TEAM safety culture model [16], several new and important challenges to safety culture are emerging, both in health care [101] and in general industry [17,102,103]. When comparing the existing TEAM safety culture model description and graphical representation [16] (p. 337) with the above findings, we observe that several elements are currently not included in the TEAM model:

- The pursuit of true safety [29,51,52], e.g., the intention to improve beyond mere legal compliance, is not explicitly mentioned the TEAM model.
- Trust, being earned by an organization in society, is of key importance. However, the term trust, as it is currently mentioned in the TEAM safety culture model, uses the phrase “Trust in the organization” in the perceptual factors area, and only applies to employees.
- The term transparency is currently placed between brackets in the TEAM model in the perceptual factors area, while its applicability is confined to internal communication between management and employees inside the organization. Using “transparency as perceived” would be more appropriate here. The impact of transparency on safety as perceived outside an organization in society is not included in the TEAM model, however.
- The TEAM model currently only describes communication health and safety inside an organization, where it mentions “transparency and open communication about safety” [16] (p. 332). Moreover, external communication about risk, health and safety are important, however.
- Assessing the organizations’ societal merit, e.g., by using a CSR performance ranking scale or a newly developed societal merit ladder, is not mentioned in the TEAM model.
- A system for knowledge sharing about health and safety outside an organization is currently not indicated in the TEAM model, although the term knowledge, applicable only to employees, is mentioned in the personal psychological factors area.
- Proactive organizational learning about health and safety, together with external stakeholders in a learning safety community, is also currently not included in the TEAM model.
- Making use of a safety information broker for safety information exchange is not mentioned in the TEAM model. Gathering information and measurement results about process and about safety and sharing this with the broker is advised.
- Measurement of safety culture inside the organization can be undertaken, e.g., via in-depth interviews, document analysis, observations and questionnaires. Measurement of the performance in the societal domain can be conducted outside the organization in society, e.g., by authorities, local communities and monitoring of shared information at a safety information broker. Together, the combined measurement results indicate the state of true safety and of the participation in a learning safety community.

### 3.7. TEAM Safety Culture Model Extension

**Figure 3:**
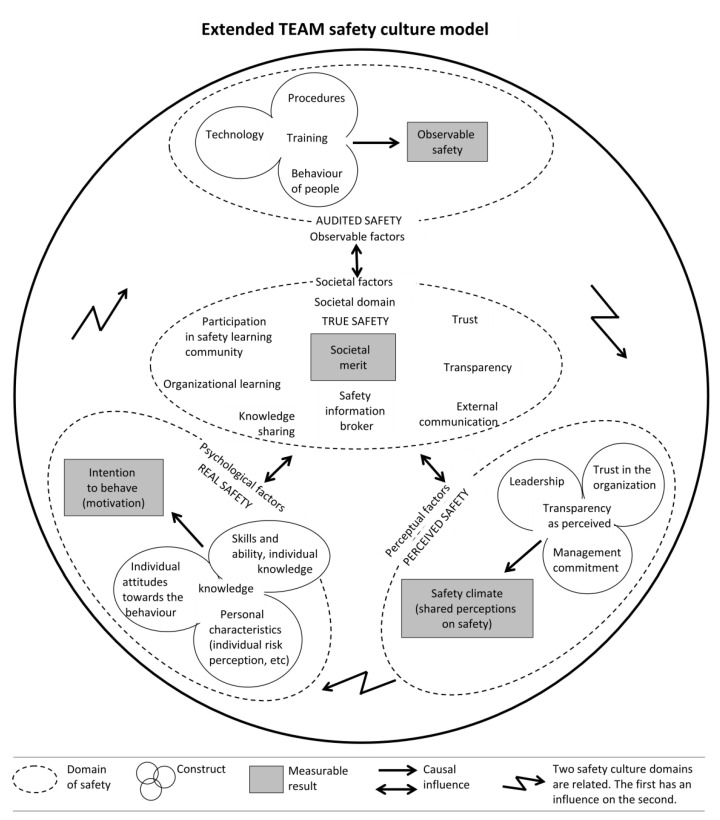
Extended TEAM safety culture model showing the proposed extension with a societal domain placed at its center.

Based on the above findings and observations from sub-question investigations, and as an answer to the main research question, the authors propose to extend the TEAM safety culture model with a fourth, societal domain at its center, see Figure 3.

In this way, the model accommodates the missing elements and addresses true safety, since the missing elements are linked for a major part to the societal position of an organization. Instead of the current name, “The Egg Aggregated Model”, the overall extended model title could best be changed to: “Extended TEAM safety culture model”. This is to better reflect its main objective: “addressing safety culture” [16] (p. 323). Rather than being closed, an organization needs to be open and actively contributing to society. This means that an unintended association with being closed or self-contained is avoided. The differing explanations of the egg protein, yolk and air presence in the original model [16,29]) are unified and placed in the footnotes. The term “real” in the dotted oval contour around observable factors [29] can best be replaced by “observable”, since “real safety” is already associated with the personal psychological factors area.

Both improvement activities on all aspects of health and safety and performance indicators are currently not explicitly indicated in the TEAM model area of audited safety. The authors regard these activities as a part of regular safety management procedures. Together, Figure 2 and Figure 3 constitute a smart health and safety transparency and information exchange framework.

### 3.8. Towards a Learning Safety Community

Learning about safety aims at prevention of accidents and harm to people. The core issue is to have the right knowledge available to “people who have the power to change outcomes” at the time and place where an accident can happen.

Knowing is based on learning. Becoming an expert in a specific setting requires work practice, learning by doing under supervision of a more experienced expert, sharing stories about experiences, imagining what could go wrong and analysis of the accident case history in order to enhance decision-making skills [14] (pp. 150–151).

Expert knowledge does not only originate from experience of people within an organization, but can also come from other organizations with a similar situation. This leads to a focus on safe work practice, shared between many organizations. Individuals within such a practice-oriented community of practice adhere to the safe work knowledge and skills, and transmit those to newcomers. This fits in the safety culture domain [56].

### 3.9. Standards Relevant to Transparency in Safety Information

Several standards are referred to in the literature [14,38,70,88,98,104] regarding transparency, health and safety. Initiatives in support of introducing transparency in standards are also mentioned [33]. None of the currently established standards explicitly address the transparency of health and safety information, however.

The authors argue that transparency should be implemented—at least—in general standards for risk management [104], corporate governance [88], Occupational Health and Safety [14], major accident prevention [98] and patient safety [38,70].

A start may be made with the standards and goals listed below:

- Risk management: ISO 31000:2018 [105],
- Corporate Governance: UNCTAD/ITE/TEB/2006/3 [106],
- Occupational safety: ISO 45001:2018 [107],
- Major Accident Prevention: “Seveso III” Directive 2012/18/EU [108],
- Patient Safety: international patient safety goals (IPSGs) [109].

## 4. Discussion

### 4.1. Limitations

This study has several limitations. The method chosen for this study makes the results vulnerable to choices made in the literature selection process, e.g., due to superficiality, which, in this case, was unavoidable because of the colossal number of returns; due to a wide variety of terms, which, in this case, was because there is no body of knowledge with unified terminology [20]; and due to a risk of author subjectivity or bias during source selection.

In spite of this, the authors contend that the two main findings—the health and safety information exchange framework (Figure 2) and the extension of the TEAM safety culture model with a societal domain (Figure 3)—will help to position transparency, organizational learning and societal merit as new development areas in safety culture practice in organizations.

### 4.2. Practical Applications

The authors propose that organizations (1) use the extended TEAM safety culture model as proposed in this study (Figure 3); then, (2) embrace transparency in their corporate policy statement; and (3) implement organizational learning in their safety management systems. The authors also propose that organizations (4) build a status measurement and monitoring tool, based on these three aspects, in support of their transparency improvement activities.

### 4.3. Recommendations

The authors recommend governments to initiate the development of a health and safety transparency standard for organizations in industry and health care, taking into account the many legal, ethical and political concerns that might be encountered.

The authors also recommend the establishment of an independent safety knowledge broker institution where all three main stakeholders can participate, contribute information and jointly take steps towards a health and safety knowledge community.

The authors recommend to further investigate the emergence of cyber-socio-technical systems and the impact on safety culture, safety management, and health and safety data exchange this may have [110].

Since the experiential knowledge residing among the general public is currently underutilized and the people can clearly be better informed about health and safety risks, the authors suggest further research to explore the possibilities for public involvement in the much-needed health, safety and wellbeing discourse in society [55]. In support of such research, the sender–medium–receiver contributions within the information flows indicated in Figure 2 can be further explored.

This study and its indicative results obtained via the scoping review approach explores the way for future studies reaching deeper into the subject matter of health and safety information transparency, organizational learning and societal merit.

## 5. Conclusions

Sharing knowledge about health and safety between the three main stakeholder groups in society can save lives and enhance wellbeing and quality of life. All three of these groups—government, organizations and the people—can benefit from transparency for their own reasons, but also have hesitations to share such knowledge since there might be damage to their interests. More transparency may be part of a solution but is not enough in isolation. The solution must be “smart transparency”. The help of a suitable safety culture model that includes the societal aspect, a standard about how to deal with transparency, an organization-wide internal safety management system which provides structure, and an overview of where health and safety information is, or should be, exchanged is necessary to develop suitable activities in practice. Currently, the government and the organizations often share less health and safety information than is desirable or needed to be a true learning community, and the people have a very limited say in health and safety matters. In the future, all three stakeholder groups can take part in a learning safety community, facilitated by a safety broker institution.
